# In Situ Formation of FeNi Nanoparticles on Polypyrrole Hydrogel for Efficient Electrocatalytic Nitrate Reduction to Ammonia

**DOI:** 10.3390/molecules30061271

**Published:** 2025-03-12

**Authors:** Lixia Li, Paihao Yan, Qinkai Guo, Dongxu Zhang, Chunliang Mao, Quan Yuan, Hongtao Sun, Mingze Liu, Yanhong Liu, Baodong Mao

**Affiliations:** 1 School of Environment and Safety Engineering, Jiangsu University, Zhenjiang 212013, China; qingpipa@ujs.edu.cn (L.L.); 2222209096@stmail.ujs.edu.cn (P.Y.);; 2 School of Chemistry and Chemical Engineering, Jiangsu University, Zhenjiang 212013, China; 3 College of Mechanical Engineering, Yanshan University, Qinhuangdao 066004, China

**Keywords:** nitrate reduction reaction, electrocatalysis, ammonia synthesis, polypyrrole hydrogel

## Abstract

The electrocatalytic reduction of nitrate to ammonia (NH_3_) under mild environmental conditions is attracting increasing attention, in which efficient and inexpensive transition metal catalysts, with the advantages of abundancy and low cost, play a key role. However, synergistic activity and selectivity promotion are still highly challenging. Herein, we developed a hydrogel-assisted strategy to prepare FeNi nanoparticles via the in situ adsorption and reduction of Fe/Ni precursors on a polypyrrole hydrogel. After optimization, the maximum NH_3_ yield reached 0.166 mmol h^−1^ cm^−2^, with a Faradaic efficiency of 88.9% and a selectivity of 86.6%. This excellent electrochemical performance was attributed to the mesoporous hydrophilic structure of the polypyrrole hydrogel, which facilitates the homogeneous loading of FeNi nanoparticles and provides a channel for both charge and mass transfer during nitrate reduction, which is important for the conversion of NO_3_^−^ to NH_3_. Electrochemical active surface area determination and impedance spectroscopy showed that the introduction of hydrogel increased the active sites and improved the charge transfer. This study provides an effective strategy for improving the selectivity and yield of NH_3_ in transition metal electrocatalysts by utilizing the three-dimensional hydrogel network and electrical conductivity.

## 1. Introduction

Ammonia (NH_3_), as a one of the most widely used chemicals, not only plays an important role in fertilizer and pharmaceuticals but also as acts as a promising environmentally friendly and carbon-free energy carrier for sustainable applications [1,2,3]. The production of NH_3_ in an industrial context mainly occurs through the Haber–Bosch process from nitrogen and hydrogen at high temperatures and pressure, which leads to greenhouse gas emission, huge energy consumption, and security problems [4,5]. Therefore, it is crucial to seek new strategies to replace traditional methods. At present, the electrocatalytic reduction of nitrogen species, such as through the nitrate reduction reaction (NO_3_RR), is as an efficient method for ammonia synthesis under mild environmental conditions [6,7,8]. The fact that NO_3_^−^ is more readily reduced to ammonia compared to N_2_ can be attributed to the lower dissociation energy of N=O bonds (204 kJ mol^−1^) [9] in contrast with the significantly higher dissociation energy of the N≡N triple bonds (941 kJ mol^−1^) [10,11]. However, the complex NO_3_RR process involves eight-electron transfer and the production of many N intermediates [12], which seriously hinders the catalytic activity and selectivity of NH_3_ [13] and poses a great challenge for developing efficient catalysts.

Currently, noble metals such as gold, ruthenium, and palladium are widely utilized for chemical detection and electrochemical NO_3_RR due to their excellent properties, but their scarcity and high cost prevent their large-scale practical application [14,15,16,17]. Therefore, cheap transition metals such as iron, nickel, and copper have been extensively researched owing to their low cost, abundancy, and unique electronic structure in order to provide active sites for NO_3_RR [18,19,20,21,22]. However, the low selectivity of NH_3_ and competitive reactions, especially hydrogen evolution, severely hinder the development of efficient transition metal catalysts [23,24,25]. To enhance NH_3_‘s selectivity and inhibit competitive reactions, new strategies need to be developed to replace the use of single transition metal catalysts. In recent years, bimetallic electrocatalytic systems have been widely reported due to their synergistic effects, which can effectively improve ammonia yield and selectivity. For example, CuCo hybrid oxides can achieve a high yield and Faraday efficiency (FE) of NH_3_ at lower potentials by taking advantage of synergistic effects and internal spin states [26]. Bimetallic FeNi electrocatalysts contribute to the transfer of nitrite and allow effective relay catalysis, thus improving selectivity during the reduction of nitrite to NH_3_ [27]. Despite already possessing the above advantages, the stability of bimetallic catalysts for NO_3_RR still needs to be further improved.

In recent years, nD (*n* = 0, 1, 2, 3) nanomaterials formed by composite metals and multidimensional materials have gradually begun to be studied owing to their unique structural properties and enhanced performance in applications such as catalysis, energy storage, and sensing [28]. A hydrogel is a polymer material with a three-dimensional mesoporous structure and great hydrophilicity. Owning to their good electrical conductivity and structural stability, hydrogels have been widely explored in the field of electrocatalysis in recent years, including for efficient water oxidation, the preparation of high-capacitance supercapacitor electrodes, and the reduction of nitrate to ammonia [29,30,31]. For example, phytic acid (PA)-based conductive hydrogels and graphene/polyaniline composite hydrogels for efficient water oxidation and high-capacitance supercapacitor electrodes have high specific capacitance and excellent cycling stability, enabling fast charge and mass transfer [32,33]. In addition, nitrogen-coordinated Fe prepared using the polymer-hydrogel method improved NH_3_ yield and FE by making the atoms on the carbon uniformly dispersed [34]. Although hydrogels have been used in electrocatalysis in recent years, more research input is still required for NO_3_RR to achieve high NH_3_ yields and high selectivity by employing the structural and electronic advantages of hydrogels.

To achieve the effective conversion of nitrate to ammonia, in this work, we prepared an iron–nickel-loaded sodium dodecyl sulfate-pyrrole (PPy) hydrogel from an electrocatalytic perspective. The resulting Fe/Ni-PPy allowed high conversion of NO_3_^−^ (93.59%) and selectivity of NH_3_ (86.6%) at −0.9 V vs. a reversible hydrogen electrode (RHE). Importantly, the yield of ammonia reached 0.166 mmol h^−1^ cm^−2^, approximately three times higher than that of the Ni-Fe catalyst, with an FE of 88.9%, indicating the high NO_3_RR activity and selectivity of this catalyst. Electrochemical active surface area (ECSA) and electrochemical impedance spectroscopy (EIS) tests indicated that the introduction of the hydrogel into the bimetallic Fe/Ni system effectively exposed more active sites and enhanced the charge transfer to improve catalytic activity. This study provides a new hydrogel-mediated strategy for the construction of bimetallic electrocatalysts for efficient ammonia production.

## 2. Results and Discussion

**Figure 1:**
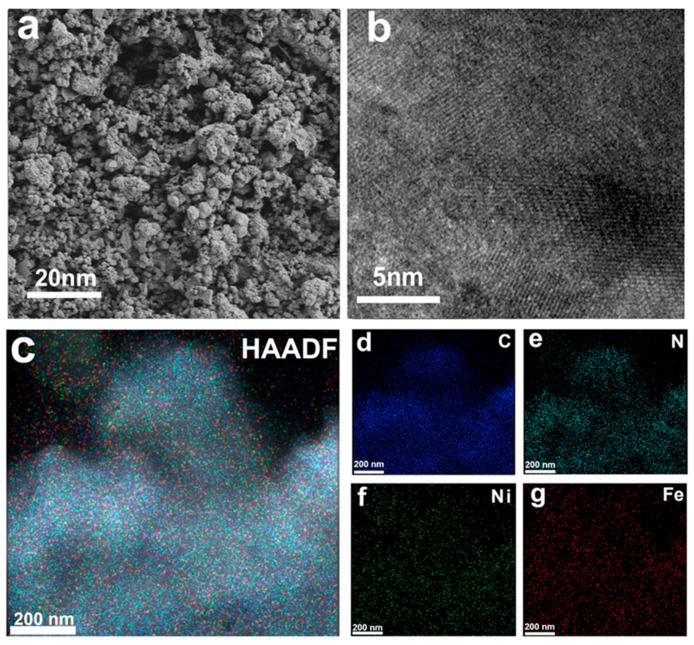
(**a**) SEM, (**b**) HRTEM, (**c**) HAADF, and (**d**–**g**) EDS mapping images of Fe/Ni−PPy.

The structure of catalysts was observed using scanning electron microscopy (SEM) and transmission electron microscopy (TEM). As shown in the SEM image in Figure 1a, the hydrogel is successfully attached to the carbon paper and the porous structure provides a larger active area for the catalyst. The TEM image of Fe/Ni-PPy (Figure S1) shows the presence of PPy loaded with FeNi nanoparticles. Meanwhile, the high-resolution TEM (HRTEM) image of Fe/Ni-PPy shows clear lattice stripes, indicating good lattice structures of the FeNi catalyst nanoparticles (Figure 1b) [35,36]. These results show that the Fe/Ni-PPy catalyst can expose more active sites, which is crucial to improve the property of the catalyst. Moreover, the corresponding element mapping images in Figure 1c–g demonstrate the presence of C, N, Ni, and Fe elements in the catalyst with a uniform distribution, further demonstrating that Fe/Ni-PPy has been successfully synthesized and uniformly attached to the carbon paper substrate [37].

**Figure 2:**
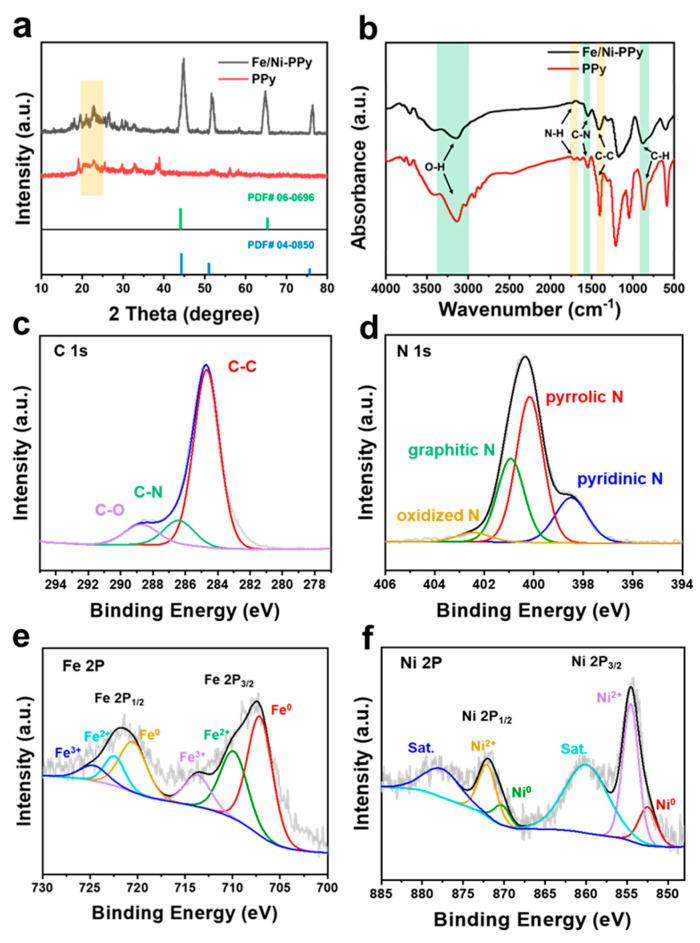
(**a**) XRD patterns (the shadows are the characteristic peaks of PPy) and (**b**) FTIR spectra of PPy and Fe/Ni−PPy. The high-resolution XPS curves of (**c**) C 1s, (**d**) N 1s, (**e**) Fe 2p, and (**f**) Ni 2p of Fe/Ni−PPy.

The crystal structure of the materials was further investigated by powder X-ray diffraction (XRD). As shown in Figure 2a, the characteristic peaks of the PPy hydrogel material are observed at 22.8° [38], and the diffraction peaks at 44.5, 51.8, and 76.4° are ascribed to the (111), (200), and (220) planes of Ni (PDF#04-0850) of Fe/Ni-PPy, respectively. The Fe/Ni-PPy also exhibits two peaks at 44.7 and 65.0°, which can be ascribed to the (110) and (200) planes of crystalline Fe (PDF#06-0696) [39]. The above results demonstrate the successful preparation of Fe/Ni-PPy [40]. Using the Debye–Scherrer formula (Formula (S1)), the average grain size of the FeNi nanoparticles in the composite was estimated to be 17.43 nm [41]. Figure 2b shows the Fourier Transform Infrared (FTIR) spectra of bare PPy and Fe/Ni-PPy, in which the absorption peaks at 1538, 1720, and 3384 cm^−1^ correspond to the five-membered C-N heterocyclic vibration, N-H stretching vibration of the pyrrole ring, and the broad band of the hydroxyl group, respectively. Compared to bare PPy, Fe/Ni-PPy exhibits an o-disubstituted C-H vibration at 790 cm^−1^ and a benzene ring C=C stretching vibration at 1479 cm^−1^ [42,43]. After the introduction of FeNi nanoparticles, the peak intensity of PPy hydrogel was significantly reduced, which was due to the coordination of Fe/Ni with the functional groups on the surface of the PPy hydrogel [44]. This also indicates the successful introduction of Fe and Ni in the hydrogel.

A comprehensive analysis of the valence state of the Fe/Ni-PPy catalyst was carried out through X-ray photoelectron spectroscopy (XPS). In Figure 2c, the binding energy of C 1s at 284.6, 286.2, and 288.3 eV belong to the characteristic peaks of C-C, C-N, and C=O structures, respectively [45,46]. The characteristic peaks at 398.7, 400.1, 401.2, and 402.7 eV of binding energy are associated with pyridinic N, pyrrolic N, graphitic N, and oxidized N, respectively (Figure 2d) [46,47]. Significantly, C and N from PPy form a variety of conjugation structures to improve the electrical conductivity of Fe/Ni-PPy, which thus enhances the intrinsic activity of the catalyst [48]. In the O 1s spectrum (Figure S2), the peaks at 531.4 and 530.2 eV correspond to the surface hydroxyl group and O_2_^2−^/O^−^ species, respectively. These species facilitate the NO_3_^−^ adsorption and proton transfer on the Fe/Ni-PPy surface, thereby enhancing the NO_3_^−^ reduction performance [49,50,51]. Meanwhile, Figure 2e shows two peaks at 710.2 and 722.3 eV, which correspond to the Fe 2p_3/2_ and 2p_1/2_ of Fe^2+^, respectively. The two peaks at 713.9 and 724.3 eV are assigned to Fe 2p_3/2_ and 2p_1/2_ of Fe^3+^, respectively. Most importantly, the peaks at 703.4 and 720.5 eV illustrate the existence of Fe^0^, which indicates that the Fe^0^ was produced in the catalyst from the reduction reactions of Fe^3+^ and Fe^2+^ during the synthesis of the material [46,52]. The XPS spectrum of Ni 2p (Figure 2f) also clearly shows the characteristic peaks of Ni^0^ at 852.8 (Ni 2p_3/2_) and 870.4 eV (Ni 2p_1/2_), while the peaks at 853.8 and 871.9 eV are assigned to Ni 2p_3/2_ and 2p_1/2_ of Ni^2+^, respectively, with two satellite peaks observed at 860.1 and 877.9 eV [53]. The above results suggest that the Fe and Ni species present in the catalyst exist in a mixed valence state, derived from the reduction reaction during the synthesis of materials. And the mixed valence states of Ni can inhibit the oxidation of Fe^0^ and ensure the stability of the catalyst [35]. The synergistic effect of Fe and Ni enhances the adsorption of nitrates and disrupts the strong correlation associated with the collaborative hydrogenation and *NH separation processes, thereby enhancing the kinetics of ammonia production [54,55].

**Figure 3:**
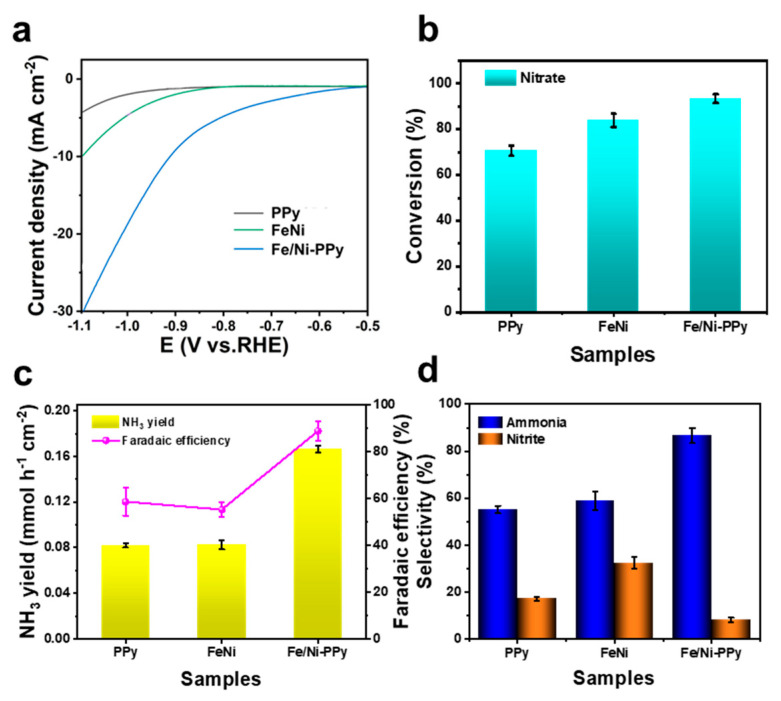
(**a**) LSV curves of PPy, FeNi, and Fe/Ni−PPy. (**b**) Conversion of NO_3_^−^, (**c**) NH_3_ yield and FE, and (**d**) selectivity of NH_3_ and NO_2_^−^ with the samples at −0.9 V vs. RHE for 1 h.

The electrochemical experiments were conducted in a Na_2_SO_4_ solution (0.5 M) with KNO_3_ (200 ppm) by using a three-electrode system. As shown in the linear sweep voltammetry (LSV) curves (Figure 3a), the Fe/Ni-PPy electrocatalyst displays an evidently lower onset potential than those of pure FeNi particles and hydrogel, indicating the excellent electrochemical properties of Fe/Ni-PPy. First, the NO_3_^−^ conversion of different samples is explored in Figure 3b, in which the Fe/Ni-PPy exhibits a higher NO_3_^−^ conversion (93.59%) at −0.9 V vs. RHE compared to PPy (70.51%) and FeNi (83.76%). Similarly, the NH_3_ yield of Fe/Ni-PPy was more than doubled compared with bimetallic FeNi particles (Figure 3c), while the FE of NH_3_ of Fe/Ni-PPy is also much higher than that of FeNi electrocatalysts. Moreover, the NO_2_^−^ selectivity of Fe/Ni-PPy is only found in a quarter of FeNi electrocatalysts (Figure 3d), indicating that PPy plays a pluripotent role in promoting the adsorption of NO_2_^−^ and its further conversion towards NH_3_. The results show that the addition of PPy provides better conditions for NO_3_RR, and the prepared Fe/Ni-PPy has better electrochemical properties than those of pure FeNi particles and PPy hydrogel electrocatalysts. This phenomenon can be attributed to the introduction of PPy, which promoted the adsorption of NO_3_^−^ and NO_2_^−^ and accelerated the conversion of intermediates to NH_3_ [56].

**Figure 4:**
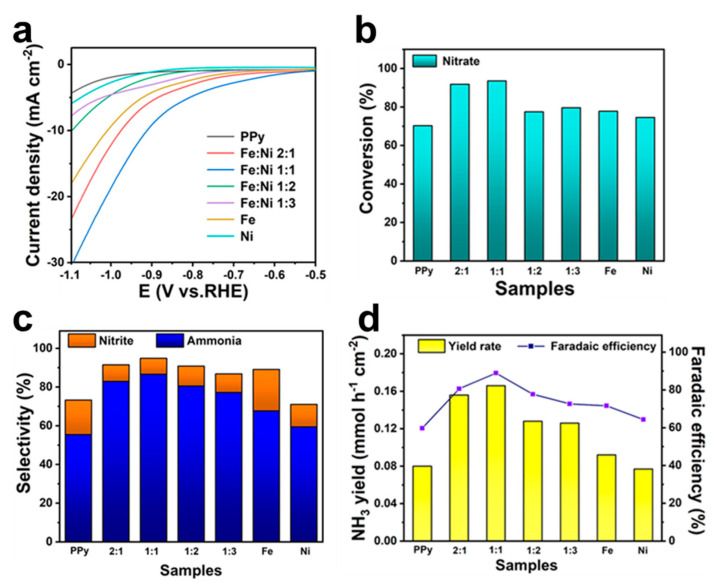
(**a**) LSV curves of different catalysts. (**b**) Conversion of NO_3_^−^, (**c**) selectivity of NH_3_ and NO_2_^−^, and (**d**) NH_3_ yield and FE with the Fe/Ni−PPy samples with different Fe:Ni ratios at −0.9 V vs. RHE for 1 h.

To investigate the optimal Fe:Ni ratio, PPy hydrogels with Fe:Ni ratios of 2:1, 1:1, 1:2, 1:3, 1:0, and 0:1 were synthesized for electrochemical tests. The current densities of different catalysts were compared by LSV curves, as shown in Figure 4a. At the same current density, the composite of FeNi with PPy can effectively reduce the onset potential of NO_3_RR. The lowest onset potential was observed at an Fe:Ni ratio of 1:1, indicating that the catalysts exhibited the highest electrochemical reaction rate under this condition. Notably, the introduction of Ni synergistically enhances the electrochemical performance with Fe, whereas an excessive amount of Ni shifts the reaction towards HER (Figure S3), thereby adversely affecting NO_3_RR [57,58]. The above observations suggest that Fe and Ni are most effective in synergizing NO_3_RR at a ratio of 1:1. At the same time, the NO_3_^−^ conversion ratio of Fe/Ni-PPy (Fe:Ni = 1:1) reaches 93.59% at −0.9 V vs. RHE, which shows an increase over 10% compared to Fe-PPy or Ni-PPy (Figure 4b). The optimized Fe/Ni-PPy catalyst also achieves the highest NH_3_ selectivity of 86.6% and the lowest NO_2_^−^ selectivity of 8.24% (Figure 4c), superior to those of pure Fe-PPy or Ni-PPy catalysts. The excellent catalyst performance is also reflected in the NH_3_ yield and FE (Figure 4d), which reaches 0.166 mmol h^−1^ cm^−2^ and 88.9%, respectively. Figure S4 shows a comprehensive and systematic comparison of Fe-PPy, Ni-PPy, PPy hydrogels, and Fe/Ni-PPy. Both the NO_3_^−^ conversion and NH_3_ selectivity of Fe/Ni-PPy were higher than those of other samples, while the selectivity of NO_2_^−^ was the lowest in all samples. These performance promotions can be ascribed to the fact that the introduction of PPy enables the uniform loading of the FeNi particles on its 3D scaffold and the fully exposed active sites.

**Figure 5:**
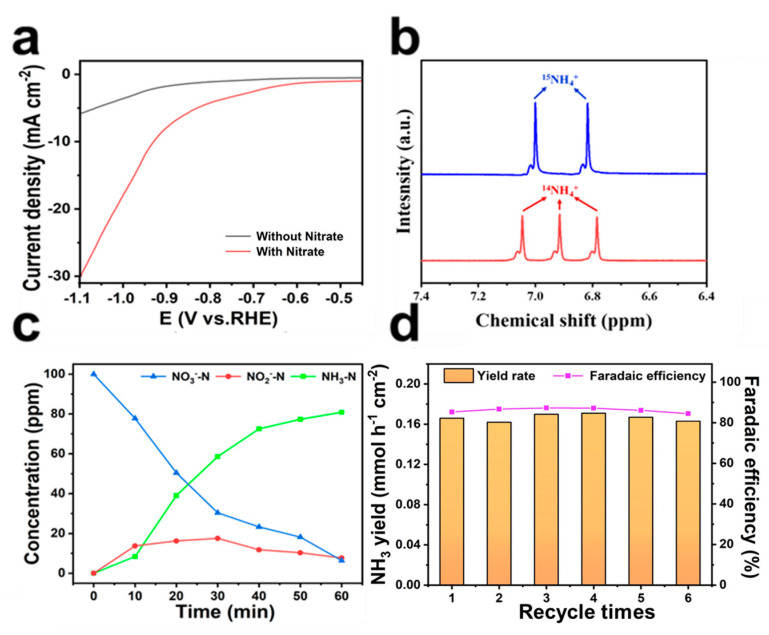
(**a**) LSV curves of Fe/Ni−PPy (Fe:Ni = 1:1) in electrolyte with or without NO_3_^−^. (**b**) The ^1^H NMR spectra of ^14^NH_4_^+^ and ^15^NH_4_^+^. (**c**) The time-dependent concentration curves of NO_3_^−^, NO_2_^−^, and NH_3_, and (**d**) the 6 consecutive cycle tests of Fe/Ni−PPy (Fe:Ni = 1:1).

To eliminate the interference of external factors and elements contained in the Fe/Ni-PPy, LSV tests were performed in the presence and absence of the NO_3_^−^ electrolyte [59]. The experimental result shows that the current density in the presence of KNO_3_ is significantly higher than the absence of KNO_3_, indicating that NO_3_^−^ is involved in the reaction (Figure 5a). To verify the source of N, a series of control experiments concerning NH_3_ yield were performed at the same potential with the conditions of open circuit, bare carbon cloth without the catalyst, and pure Na_2_SO_4_ electrolyte without NO_3_^−^. The results show that the N source is exclusively from NO_3_^−^ in the electrolyte, with no contribution from pollutions in the environment or the catalyst (Figure S5). Under different voltages, the NO_3_^−^ conversion test (Figure S6) indicates a rapid increase in the conversion rate from 74.68% to 93.59% from −0.7 to −0.9 V vs. RHE, followed by a gradual decrease. The varying trend of the selectivity of NO_2_^−^ and NH_3_ indicates that NO_3_^−^ gradually transforms into NH_3_ at high voltage and the selectivity of NO_2_^−^ decreases at −0.7 to −1.1 V vs. RHE (Figure S7). It is noteworthy that the selectivity of NH_3_ undergoes a slight decrease as a result of the hydrogel structure being destructively affected by the high potential [60]. In order to determine the source of nitrogen, K^15^NO_3_ and K^14^NO_3_ were used as nitrogen sources. The ^1^H nuclear magnetic resonance (NMR) spectra (Figure 5b) showed characteristic double peaks of ^15^NH_4_^+^ and triple peaks of ^14^NH_4_^+^. It was proved that the NH_4_^+^ product was from the electrocatalytic reduction of NO_3_^−^ by Fe/Ni-PPy, and the measured NH_3_ concentration is reliable and not contaminated. The volcanic curve in Figure S8 shows that the yield of NH_3_ first rises and then falls from −0.7 to −1.1 V vs. RHE. However, competitive hydrogen evolution reactions at high potentials result in lower NH_3_ yield and FE [61]. The time-dependent concentration curves depict the variations in NO_3_^−^, NO_2_^−^, and NH_3_ throughout the NO_3_RR process (Figure 5c), in which the NH_3_ concentration continues to increase and the concentration of NO_3_^−^ steadily decreases and NO_2_^−^ increases within the first 30 min and then gradually decreases, indicating that the Fe/Ni-PPy possesses the ability to accelerate the conversion of NO_2_^−^ to NH_3_. Additionally, the NH_3_ yield reached 0.166 mmol h^−1^ cm^−2^ and the FE was stable above 80% throughout six consecutive tests at −0.9 V vs. RHE (Figure 5d), suggesting the outstanding stability of Fe/Ni-PPy. Furthermore, the structure and morphology of Fe/Ni-PPy can be well reserved after the stability test, as evidenced by TEM (Figure S9). All measurements indicate that Fe/Ni-PPy possesses good structural stability. In conclusion, post-test analyses confirm that Fe/Ni-PPy exhibits adequate chemical stability for NO_3_RR, thereby establishing a solid basis for the resource utilization of NO_3_^−^.

**Figure 6:**
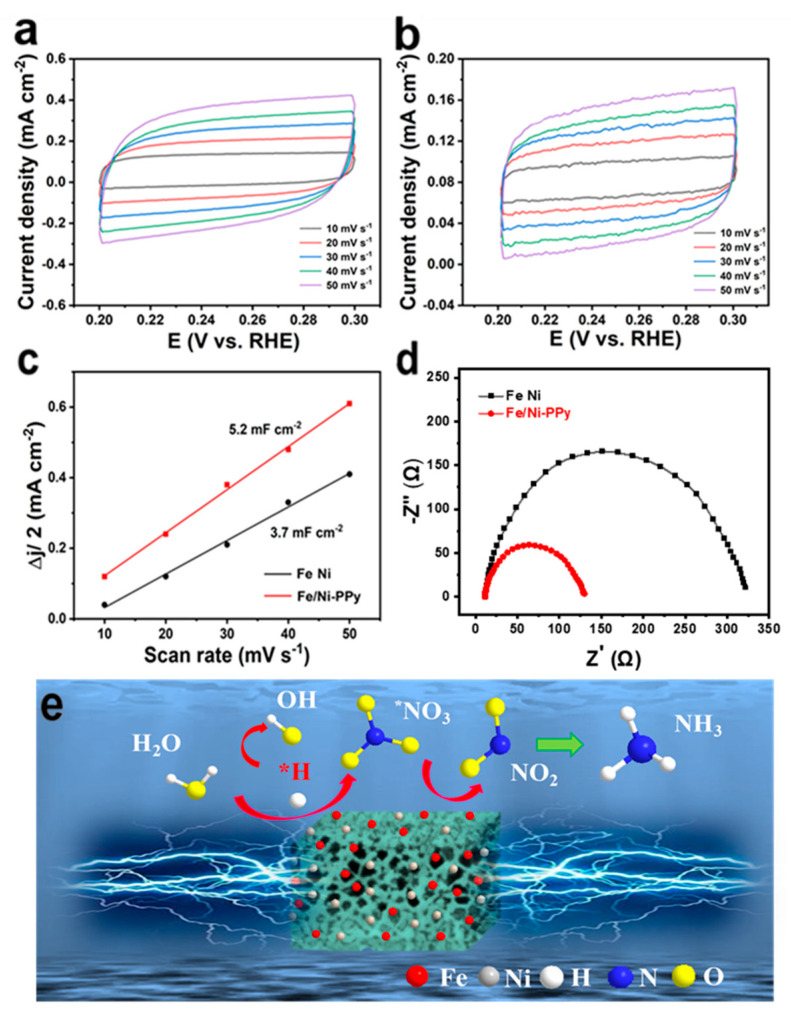
CV curves with different scan rates of (**a**) Fe/Ni−PPy and (**b**) FeNi nanoparticles. (**c**) C_dl_ diagram fitted from the CV curves. (**d**) EIS patterns of FeNi and Fe/Ni−PPy. (**e**) Schematic diagram of NO_3_RR on Fe/Ni−PPy (*H and *NO_3_ are the hydrogen atoms and NO_3_^−^ adsorbed on the catalyst surface, respectively).

To explore the intrinsic reasons for the outstanding catalytic performance of the Fe/Ni-PPy electrocatalysts, the ECSA was calculated using the electrochemical double-layer capacitance (C_dl_) method. The cyclic voltammetry (CV) tests (Figure 6a,b) were performed on Fe/Ni-PPy and bimetallic FeNi electrocatalysts at different scan rates within the potential range of 0.2–0.3 V vs. RHE. The results (Figure 6c) show that Fe/Ni-PPy reached 5.2 mF cm^−2^, while FeNi nanoparticles reached 3.7 mF cm^−2^, indicating a larger ECSA for Fe/Ni-PPy. To further evaluate the impact of PPy on the kinetics during the reaction process, EIS measurements of FeNi nanoparticles and Fe/Ni-PPy were carried out under the open circuit potential. As shown in Figure 6d, FeNi nanoparticles display a large impedance arc, which reveals the slow electrochemical behaviors of the sample. In contrast, Fe/Ni-PPy exhibits a much smaller resistance arc, indicating that the addition of the conductive PPy hydrogel can efficiently reduce the charge transfer resistance and enhance the charge transfer rate [62]. The above results illustrate that introducing PPy enhances the NO_3_^−^ reduction ability and accelerates the charge transfer in the NO_3_RR process.

Therefore, based on the above experiments and characterizations, a plausible mechanism for the NO_3_RR on Fe/Ni-PPy is proposed in Figure 6e. With Fe/Ni-PPy and the applied bias, NO_3_^−^ is transformed into *NO_3_, which undergoes hydrogenation with *H atoms from water to form NO_2_^−^. Eventually, NO_2_^−^ undergoes further sequential hydrogenation to produce NH_3_. The synergy of Fe and Ni enables low energy barriers for NH_3_ production, while the combination of the conductive hydrogel with the bimetallic FeNi nanoparticles can effectively expose more active sites and enhance the charge collection to improve the catalytic activity. This study provides a useful hydrogel-mediated strategy for the construction of composite electrocatalysts for efficient ammonia production.

## 3. Experimental Section

### 3.1. Chemicals and Materials

Sodium dodecyl sulfate (SDS, C_12_H_25_SO_4_Na), pyrrole (C_4_H_5_N), potassium nitrate (KNO_3_), sodium nitrite (NaNO_2_), ammonium persulfate ((NH_4_)_2_S_2_O_8_), ferric chloride hexahydrate (FeCl_3_·6H_2_O), nickel chloride hexahydrate (NiCl_2_·6H_2_O), sodium borohydride (NaBH_4_), sodium sulfate (Na_2_SO_4_), hydrochloric acid (HCl), salicylic acid (C_7_H_6_O_3_), sodium citrate dehydrate (C_6_H_5_Na_3_O_7_), sodium hydroxide (NaOH), sodium hypochlorite (NaClO), sulfamic acid (H_3_NO_3_S), sulfanilamide (C_6_H_8_N_2_O_2_S), N-(1-naphthyl) ethyldiamine dihydrochloride (C_12_H_14_N_2_·2HCl), and phosphoric acid (H_3_PO_4_) were purchased from Sinopharm Group Chemical Reagent Co., Ltd., Shanghai, China. Ethanol (C_2_H_5_OH) and sodium nitroprusside (C_5_FeN_6_Na_2_O·2H_2_O) were purchased from Shanghai Aladdin Bio-Chem Technology Co., Ltd., Shanghai, China. The Nafion film was supplied by the Du Pont China Holding Co., Ltd., Shanghai, China.

### 3.2. Synthesis of the Catalysts

Pyrrole (416 μL) and SDS (0.58 g) were added to 10 mL of deionized water and stirred for 40 min until a homogeneous solution was obtained (solution A). Meanwhile, 1.5 g of (NH_4_)_2_S_2_O_8_ was added into 10 mL of deionized water and sonicated until dissolved (solution B). The two solutions were sonicated and then mixed to form a black solution, which was left to stand for 1 h. After the polymerization reaction, the unreacted monomer was washed off with deionized water to obtain the PPy hydrogel.

For Fe/Ni-PPy, FeCl_3_ (0.05 M) and NiCl_2_ (0.05 M) were dissolved in deionized water (40 mL), sonicated for 5 min, and poured into the prepared hydrogel to form a mixed solution, which was stirred for 10 h to allow the adsorption of the metal ions. At the end of the process, Fe/Ni-PPy with an Fe:Ni ratio of 1:1 was obtained by adding the NaBH_4_ solution dropwise under nitrogen atmosphere to reduce the metal salts to FeNi nanoparticles on PPy. It was washed three times with deionized water and ethanol, and then dried in a vacuum oven at 40 °C for 12 h, and then ground into a powder and stored at room temperature. The Fe/Ni-PPy samples with different Fe/Ni ratios (1:3, 1:2, 2:1, 1:0, and 0:1) were also prepared using this method.

Pure FeNi nanoparticles were also prepared by using the same procedure but without PPy.

For the preparation of the working electrode, rectangular sheets of carbon paper (0.5 cm × 2 cm) were pretreated with 0.5 M sulfuric acid for 4 h, and then rinsed with deionized water. The Fe/Ni-PPy powder (10 mg) was mixed ultrasonically with the prepared solution (980 µL of ethanol and 20 µL of 5% Nafion solution). Then, 20 µL of the mixture was dropped on both sides of the carbon paper and dried naturally for further use.

### 3.3. Material Characterizations

The morphology of the samples was analyzed through a scanning electron microscope (SEM, JSM-7800F, JEOL, Tokyo, Japan) at 20 kV voltage. Transmission electron microscopy (TEM), high-resolution transmission electron microscopy (HRTEM) and energy dispersive X-ray spectroscopy (EDS) were tested using a Tecnai G2 F30 S-Twin microscope (FEI, Hillsboro, OR, USA). With a D/MAX-2500 diffractometer (Rigaku, Tokyo, Japan), X-ray diffraction (XRD) patterns were obtained utilizing Cu Kα radiation (λ = 1.5406 Å) as the source, with a 2θ angle range of 5–80°, operated at 40 kV and 30 mA. Fourier Transform Infrared (FTIR) spectroscopy was tested by Nexus 470 (Nicolet, Waltham, MA, USA). The chemical valence and composition of the catalysts were determined by X-ray photoelectron spectroscopy (XPS, Thermo Scientific ESCALAB 250X, Waltham, MA, USA), and the absorbance of the products was quantified by an ultraviolet visible spectrophotometer (Shimadzu UV2600, Kyoto, Japan). The isotopic labeling experiments were measured by ^1^H nuclear magnetic resonance (NMR) analysis (AC-P400, BRUKER, Baden-Württemberg, Germany).

### 3.4. Electrocatalytic Measurements

All electrochemical experiments were conducted utilizing a CHI 760E electrochemical workstation (Chenhua, Shanghai, China). The electrocatalytic reaction system consists of a three-electrode structure separated by a Nafion 117 membrane and an H-type electrolytic cell. The Fe/Ni-PPy loaded on carbon paper, Ag/AgCl, and graphite rod were used as the working, reference, and counter electrodes, respectively. As an electrolyte, 70 mL of 0.5 M Na_2_SO_4_ solution was added to the anode chamber, and 70 mL of 0.5 M Na_2_SO_4_ containing 200 ppm KNO_3_ was introduced to the cathode chamber, serving as the nitrogen source. The primary electrochemical evaluations encompass linear sweep voltammetry (LSV), electrochemical impedance spectroscopy (EIS), and cyclic voltammetry (CV). The EIS measurements were conducted in the frequency range of 0.1  to 1 MHz at the open circuit potential [63]. Argon was introduced before the experiment to eliminate other gas interference. The reaction was performed at a stirring rate of 350 rpm for 1 h at different potentials. The performance calculation formulae are shown in Equations (1)–(5).

(1) $${{\mathrm{NO}}_{3}}^{-}\;\mathrm{conversion}\;(\%)=(∆c_{N{O_{3}}^{-}}/c_{0})\times 100\%$$

(2) $${{\mathrm{NO}}_{2}}^{-}\;\mathrm{selectivity}\;(\%)=(c_{N{O_{2}}^{-}}/46)/(\Delta c_{N{O_{3}}^{-}}/62)\times 100\%$$

(3) $${\mathrm{NH}}_{3}\;\mathrm{selectivity}\;(\%)=(c_{NH_{3}}/17)/(\Delta c_{N{O_{3}}^{-}}/62)\times 100\%$$

(4) $$\mathrm{Yield}\;\mathrm{of}\;{\mathrm{NH}}_{3}\;(\mathrm{mmol}\;h^{-1}\;{\mathrm{cm}}^{-2})=(c_{NH_{3}}\times V)/(M_{NH_{3}}\times S\times t)$$

(5) $$\mathrm{Faradaic}\;\mathrm{efficiency}\;(\%)\;=\;\left(8\times F\times c_{NH_{3}}\times V\right)/(M_{NH_{3}}\times Q)$$

Δ*c_NO_*_3_^−^(mg L^−1^) is the concentration difference before and after the NO_3_RR reaction and *c*_0_ is the initial concentration of nitrate (mg L^−1^). The *c_NO_*_2_^−^ and *c_NH_*_3_ are the measured concentrations of NO_2_^−^ and NH_3_, respectively. *V* is the volume of the electrolyte in the electrolytic cell (70 mL). *S* is the electrode area (1 cm^2^). *t* is the electrolysis time (1 h). *F* is the Faraday constant (96,485 C mol^−1^). *Q* is the total charge through the electrode (C). *M_NH_*_3_ is the relative molecular mass of NH_3_ [64].

NO_3_^−^, NO_2_^−^, and NH_3_ were determined by different chromogenic reagents. The chromogenic reagents of NO_3_^−^ are HCl and H_3_NO_3_S. The chromogenic reagents of NO_2_^−^ are a mixed solution of C_12_H_14_N_2_·2HCl, C_6_H_8_N_2_O_2_S, and H_3_PO_4_ [65]. The concentration of NH_3_ was determined by the indophenol blue method [66]. Supporting Information shows the specific details of all the methods. Finally, the standard curves were obtained by drawing the relationship between the absorbance and the measured objects (Figures S10–S12).

## 4. Conclusions

In this paper, a series of Fe/Ni-PPy electrocatalysts with varying Fe/Ni ratios were constructed by using the PPy hydrogel as a scaffold for the in situ adsorption and formation of FeNi nanoparticles, which shows promoted NH_3_ yield and FE in NO_3_RR. The results show that, with optimized Fe:Ni = 1:1, the Fe/Ni-PPy catalyst has an NH_3_ yield of 0.166 mmol h^−1^ cm^−2^ and a FE of 88.9% at −0.9 V vs. RHE. Moreover, the ECSA and EIS indicate that the combination of FeNi bimetallic nanoparticles and PPy can effectively expose more active sites and enhance the charge transfer to improve the catalytic activity and selectivity. The mesoporous hydrophilic structure of the PPy hydrogel enables Fe and Ni to be uniformly dispersed and provides a favorable environment for bimetallic synergism, which enhances the ability of the catalysts to convert NO_3_^−^ to NH_3_. By further analyzing the N-species variation during electrolysis, it was found that Fe/Ni-PPy accelerates the rate-limiting step of NO_3_^−^ to NO_2_^−^ during NO_3_RR, owing to the improved catalyst particle loading and substrate adsorption with the introduction of the hydrogel. This study provides an interesting inspiration for the development of hydrogel-based electrocatalysts for NO_3_RR.

## Data Availability

Data will be made available on request.

## Supplementary Materials

The following supporting information can be downloaded at https://www.mdpi.com/article/10.3390/molecules30061271/s1, Figure S1: TEM image of Fe/Ni-PPy (Fe:Ni = 1:1); Figure S2: XPS spectrum of O 1s; Figure S3: LSV curves of different catalysts with 0.5 M Na_2_SO_4_; Figure S4: The performance comparison of different catalysts at −0.9 V vs. RHE for 1 h; Figure S5: NH_3_ yield in different conditions; Figure S6: The conversion of NO_3_^−^ by the Fe/Ni-PPy (Fe:Ni=1:1) at different potentials; Figure S7: The selectivity of NH_3_ and NO_2_^−^ by the Fe/Ni-PPy (Fe:Ni=1:1) catalyst at different potentials; Figure S8: The NH_3_ yield and FE by the Fe/Ni-PPy (Fe:Ni=1:1) catalyst at different potentials; Figure S9: TEM image of Fe/Ni-PPy (Fe:Ni=1:1) after 6 continuous cycle tests; Figure S10: Standard curve of NO_3_^−^; Figure S11: Standard curve of NO_2_^−^; Figure S12: Standard curve of NH_3_.

## References

[1] Yüzbaşıoğlu A.E., Avşar C., Gezerman A.O. (2022). The current situation in the use of ammonia as a sustainable energy source and its industrial potential. Curr. Res. Green Sustain. Chem..

[2] Xu Y., Ma Y., Cayuela M.L., Sánchez-Monedero M.A., Wang Q. (2020). Compost biochemical quality mediates nitrogen leaching loss in a greenhouse soil under vegetable cultivation. Geoderma.

[3] Xu Z., Zhong S., Yu Y., Wang Y., Cheng H., Du D., Wang C. (2023). *Rhus typhina* L. triggered greater allelopathic effects than Koelreuteria paniculata Laxm under ammonium fertilization. Sci. Hortic..

[4] Ren Y., Li S., Yu C., Zheng Y., Wang C., Qian B., Wang L., Fang W., Sun Y., Qiu J. (2024). NH_3_ Electrosynthesis from N_2_ Molecules: Progresses, Challenges, and Future Perspectives. J. Am. Chem. Soc..

[5] Wei W., Hu X., Yang S., Wang K., Zeng C., Hou Z., Cui H., Liu S., Zhu L. (2022). Denitrifying halophilic archaea derived from salt dominate the degradation of nitrite in salted radish during pickling. Food Res. Int..

[6] Guo X., Du H., Qu F., Li J. (2019). Recent progress in electrocatalytic nitrogen reduction. J. Mater. Chem. A.

[7] Dai H., Liu L., Zhao H., Zhou P., Ying Y., Yin M., Wang X., Fan W., Bai H. (2024). Efficient electrocatalytic reduction of nitrate to ammonia using Cu–CeO_2_ solid solution. Int. J. Hydrogen Energy.

[8] Estudillo-Wong L.A., Santillán-Díaz G., Arce-Estrada E.M., Alonso-Vante N., Manzo-Robledo A. (2013). Electroreduction of NOxz species in alkaline medium on Pt nanoparticles. Electrochim. Acta.

[9] Yao J., Yan J. (2020). Efficient nitrate-to-ammonia transformation through a direct eight-electron reduction. Sci. China Chem..

[10] Tang C., Qiao S.-Z. (2019). How to explore ambient electrocatalytic nitrogen reduction reliably and insightfully. Chem. Soc. Rev..

[11] Cui X., Tang C., Zhang Q. (2018). A Review of Electrocatalytic Reduction of Dinitrogen to Ammonia under Ambient Conditions. Adv. Energy Mater..

[12] Wang Y., Yu Y., Jia R., Zhang C., Zhang B. (2019). Electrochemical synthesis of nitric acid from air and ammonia through waste utilization. Natl. Sci. Rev..

[13] Zeng Y., Priest C., Wang G., Wu G. (2020). Restoring the Nitrogen Cycle by Electrochemical Reduction of Nitrate: Progress and Prospects. Small Methods.

[14] Peng W., Luo M., Xu X., Jiang K., Peng M., Chen D., Chan T.-S., Tan Y. (2020). Spontaneous Atomic Ruthenium Doping in Mo_2_CTX MXene Defects Enhances Electrocatalytic Activity for the Nitrogen Reduction Reaction. Adv. Energy Mater..

[15] Sun L., Liu B. (2023). Mesoporous PdN Alloy Nanocubes for Efficient Electrochemical Nitrate Reduction to Ammonia. Adv. Mater..

[16] Han E., Li L., Gao T., Pan Y., Cai J. (2024). Nitrite determination in food using electrochemical sensor based on self-assembled MWCNTs/AuNPs/poly-melamine nanocomposite. Food Chem..

[17] Wu H., Xie R., Hao Y., Pang J., Gao H., Qu F., Tian M., Guo C., Mao B., Chai F. (2023). Portable smartphone-integrated AuAg nanoclusters electrospun membranes for multivariate fluorescent sensing of Hg^2+^, Cu^2+^ and l-histidine in water and food samples. Food Chem..

[18] Gibert O., Abenza M., Reig M., Vecino X., Sánchez D., Arnaldos M., Cortina J.L. (2022). Removal of nitrate from groundwater by nano-scale zero-valent iron injection pulses in continuous-flow packed soil columns. Sci. Total Environ..

[19] Wang X., Pan Y., Wang X., Guo Y., Ni C., Wu J., Hao C. (2022). High performance hybrid supercapacitors assembled with multi-cavity nickel cobalt sulfide hollow microspheres as cathode and porous typha-derived carbon as anode. Ind. Crops Prod..

[20] Iarchuk A., Dutta A., Broekmann P. (2022). Novel Ni foam catalysts for sustainable nitrate to ammonia electroreduction. J. Hazard. Mater..

[21] Zhu T., Chen Q., Liao P., Duan W., Liang S., Yan Z., Feng C. (2020). Single-Atom Cu Catalysts for Enhanced Electrocatalytic Nitrate Reduction with Significant Alleviation of Nitrite Production. Small.

[22] Wu Z., Song Y., Guo H., Xie F., Cong Y., Kuang M., Yang J. (2024). Tandem catalysis in electrocatalytic nitrate reduction: Unlocking efficiency and mechanism. Interdiscip. Mater..

[23] Chen G.-F., Yuan Y., Jiang H., Ren S.-Y., Ding L.-X., Ma L., Wu T., Lu J., Wang H. (2020). Electrochemical reduction of nitrate to ammonia via direct eight-electron transfer using a copper–molecular solid catalyst. Nat. Energy.

[24] Carvalho O.Q., Marks R., Nguyen H.K.K., Vitale-Sullivan M.E., Martinez S.C., Árnadóttir L., Stoerzinger K.A. (2022). Role of Electronic Structure on Nitrate Reduction to Ammonium: A Periodic Journey. J. Am. Chem. Soc..

[25] Wang Y., Zhou W., Jia R., Yu Y., Zhang B. (2020). Unveiling the Activity Origin of a Copper-based Electrocatalyst for Selective Nitrate Reduction to Ammonia. Angew. Chem. Int. Ed..

[26] Sun S., Dai C., Zhao P., Xi S., Ren Y., Tan H.R., Lim P.C., Lin M., Diao C., Zhang D. (2024). Spin-related Cu-Co pair to increase electrochemical ammonia generation on high-entropy oxides. Nat. Commun..

[27] Ma X., Zhong J., Huang W., Wang R., Li S., Zhou Z., Li C. (2023). Tuning the d-band centers of bimetallic FeNi catalysts derived from layered double hydroxides for selective electrocatalytic reduction of nitrates. Chem. Eng. J..

[28] Estudillo-Wong L.A., Guerrero-Barajas C., Vázquez-Arenas J., Alonso-Vante N. (2023). Revisiting Current Trends in Electrode Assembly and Characterization Methodologies for Biofilm Applications. Surfaces.

[29] Gao D., Fabiano S. (2024). Conductive hydrogels put electrons in charge. Science.

[30] Li P., Sun W., Li J., Chen J.-P., Wang X., Mei Z., Jin G., Lei Y., Xin R., Yang M. (2024). N-type semiconducting hydrogel. Science.

[31] Zhang H., Gan X., Yan Y., Zhou J. (2024). A Sustainable Dual Cross-Linked Cellulose Hydrogel Electrolyte for High-Performance Zinc-Metal Batteries. Nano Micro Lett..

[32] Hu Q., Li G., Liu X., Zhu B., Chai X., Zhang Q., Liu J., He C. (2019). Superhydrophilic Phytic-Acid-Doped Conductive Hydrogels as Metal-Free and Binder-Free Electrocatalysts for Efficient Water Oxidation. Angew. Chem. Int. Ed..

[33] Ji J., Li R., Li H., Shu Y., Li Y., Qiu S., He C., Yang Y. (2018). Phytic acid assisted fabrication of graphene/polyaniline composite hydrogels for high-capacitance supercapacitors. Compos. Part B.

[34] Li P., Jin Z., Fang Z., Yu G. (2021). A single-site iron catalyst with preoccupied active centers that achieves selective ammonia electrosynthesis from nitrate. Energy Environ. Sci..

[35] Shan A., Idrees A., Zaman W.Q., Mohsin A., Abbas Z., Stadler F.J., Lyu S. (2024). Synthesis of CaCO_3_ supported nano zero-valent iron-nickel nanocomposite (nZVI-Ni@CaCO_3_) and its application for trichloroethylene removal in persulfate activated system. Environ. Res..

[36] Muthusamy T., Sethuram Markandaraj S., Shanmugam S. (2022). Nickel nanoparticles wrapped in N-doped carbon nanostructures for efficient electrochemical reduction of NO to NH_3_. J. Mater. Chem. A.

[37] Wang C., Yang H., Zhang Y., Wang Q. (2019). NiFe Alloy Nanoparticles with hcp Crystal Structure Stimulate Superior Oxygen Evolution Reaction Electrocatalytic Activity. Angew. Chem. Int. Ed..

[38] Zheng H., Chen M., Sun Y., Zuo B. (2022). Self-Healing, Wet-Adhesion silk fibroin conductive hydrogel as a wearable strain sensor for underwater applications. Chem. Eng. J..

[39] Yang Y., Xu Y., Zhong D., Qiao Q., Zeng H. (2024). Efficient removal of Cr(VI) by chitosan cross-linked bentonite loaded nano-zero-valent iron composite: Performance and mechanism. J. Hazard. Mater..

[40] Vigneshwaran J., Jose J., Thomas S., Gagliardi A., Narayan R.L., Jose S.P. (2024). PPy-PdO modified MXene for flexible binder-free electrodes for asymmetric supercapacitors: Insights from experimental and DFT investigations. Chem. Eng. J..

[41] Khalil K.D., Riyadh S.M., Alkayal N.S., Bashal A.H., Alharbi K.H., Alharbi W. (2022). Chitosan-Strontium Oxide Nanocomposite: Preparation, Characterization, and Catalytic Potency in Thiadiazoles Synthesis. Polymers.

[42] Chen S., Guo B., Yu J., Yan Z., Liu R., Yu C., Zhao Z., Zhang H., Yao F., Li J. (2024). A polypyrrole-dopamine/poly(vinyl alcohol) anisotropic hydrogel for strain sensor and bioelectrodes. Chem. Eng. J..

[43] Guan D., Xu H., Huang Y.-C., Jing C., Tsujimoto Y., Xu X., Lin Z., Tang J., Wang Z., Sun X. (2024). Operando Studies Redirect Spatiotemporal Restructuration of Model Coordinated Oxides in Electrochemical Oxidation. Adv. Mater..

[44] Wang J., Du P., Hsu Y.-I., Uyama H. (2024). Smart versatile hydrogels tailored by metal-phenolic coordinating carbon and polypyrrole for soft actuation, strain sensing and writing recognition. Chem. Eng. J..

[45] Zhang Y., Chen L., Yan B., Zhang F., Shi Y., Guo X. (2023). Single Cu atoms confined in N-doped porous carbon networks by flash nanocomplexation as efficient trifunctional electrocatalysts for Zn-air batteries and water splitting. Compos. Part B.

[46] Wang Z., Ang J., Liu J., Ma X.Y.D., Kong J., Zhang Y., Yan T., Lu X. (2020). FeNi alloys encapsulated in N-doped CNTs-tangled porous carbon fibers as highly efficient and durable bifunctional oxygen electrocatalyst for rechargeable zinc-air battery. Appl. Catal. B.

[47] Guo Y., Yao S., Gao L., Chen A., Jiao M., Cui H., Zhou Z. (2020). Boosting bifunctional electrocatalytic activity in S and N co-doped carbon nanosheets for high-efficiency Zn–air batteries. J. Mater. Chem. A.

[48] Tang S., Yu C., Liu X., Fu D., Liao W., Xu F., Zhong W. (2022). 2-Methylimidazole assisted synthesis of nanocrystalline shell reinforced PPy hydrogel with high mechanical and electrochemical performance. Chem. Eng. J..

[49] Cai J., Huang J., Cao A., Wei Y., Wang H., Li X., Jiang Z., Waterhouse G.I.N., Lu S., Zang S.-Q. (2023). Interfacial hydrogen bonding-involved electrocatalytic ammonia synthesis on OH-terminated MXene. Appl. Catal. B.

[50] Abdelghafar F., Xu X., Guan D., Lin Z., Hu Z., Ni M., Huang H., Bhatelia T., Jiang S.P., Shao Z. (2024). New Nanocomposites Derived from Cation-Nonstoichiometric Bax(Co, Fe, Zr, Y)O_3−δ_ as Efficient Electrocatalysts for Water Oxidation in Alkaline Solution. ACS Mater. Lett..

[51] Gong Z., Zhong W., He Z., Liu Q., Chen H., Zhou D., Zhang N., Kang X., Chen Y. (2022). Regulating surface oxygen species on copper (I) oxides via plasma treatment for effective reduction of nitrate to ammonia. Appl. Catal. B.

[52] Huang C.-C., Pourzolfaghar H., Huang C.-L., Liao C.-P., Li Y.-Y. (2024). FeNi nanoalloy-carbon nanotubes on defected graphene as an excellent electrocatalyst for lithium-oxygen batteries. Carbon.

[53] Zhang C., Gao Y., Zhang J., Jiao Y., Zhu Q., Wang J., Chen Y., Li X. (2025). Regulating Ni microchemical state for enhanced C-C/H bonds adsorption and activation towards methylcyclohexane steam reforming. Chem. Eng. J..

[54] Ma L., Xu F., Zhang L., Nie Z., Xia K., Guo M., Li M., Ding X. (2022). Breaking the linear correlations for enhanced electrochemical nitrogen reduction by carbon-encapsulated mixed-valence Fe_7_(PO_4_)_6_. J. Energy Chem..

[55] Qiao H., Han Y., Yao L., Xu X., Ma J., Wen B., Hu J., Huang H. (2023). Coating zero valent iron onto hollow carbon spheres as efficient electrocatalyst for N_2_ fixation and neutral Zn-N_2_ battery. Chem. Eng. J..

[56] Chen H., Zhang Z., Li Y., Yu L., Chen F., Li L. (2024). Conductive polymer protection strategy to promote electrochemical nitrate reduction to ammonia in highly acidic condition over Cu-based catalyst. Chem. Eng. J..

[57] Luo H., Li S., Wu Z., Liu Y., Luo W., Li W., Zhang D., Chen J., Yang J. (2023). Modulating the Active Hydrogen Adsorption on Fe–N Interface for Boosted Electrocatalytic Nitrate Reduction with Ultra-Long Stability. Adv. Mater..

[58] Xiong Y., Sun M., Wang S., Wang Y., Zhou J., Hao F., Liu F., Yan Y., Meng X., Guo L. (2024). Atomic Scale Cooperativity of Alloy Nanostructures for Efficient Nitrate Electroreduction to Ammonia in Neutral Media. Adv. Funct. Mater..

[59] Kou M., Yuan Y., Zhao R., Wang Y., Zhao J., Yuan Q., Zhao J. (2024). Insights into the Origin of Activity Enhancement via Tuning Electronic Structure of Cu_2_O towards Electrocatalytic Ammonia Synthesis. Molecules.

[60] Deng K., Lian Z., Wang W., Yu J., Mao Q., Yu H., Wang Z., Wang L., Wang H. (2024). Hydrogen spillover effect tuning the rate-determining step of hydrogen evolution over Pd/Ir hetero-metallene for industry-level current density. Appl. Catal. B Environ. Energy.

[61] Niu Z., Fan S., Li X., Wang P., Liu Z., Wang J., Bai C., Zhang D. (2022). Bifunctional copper-cobalt spinel electrocatalysts for efficient tandem-like nitrate reduction to ammonia. Chem. Eng. J..

[62] Mondal S., Dilly Rajan K., Patra L., Rathinam M., Ganesh V. (2025). Sulfur Vacancy-Induced Enhancement of Piezocatalytic H_2_ Production in MoS_2_. Small.

[63] Zhang X., Kohler H., Schwotzer M., Guth U. (2021). Stability improvement of layered Au,Pt-YSZ mixed-potential gas sensing electrodes by cathodic polarization: Studies by steady state and dynamic electrochemical methods. Sens. Actuators B.

[64] Zhang D., Liu Y., Li D., Jiang T., Chen Q., Mao C., Li L., Jiang D., Mao B. (2025). Carbon dots-boosted active hydrogen for efficient electrocatalytic reduction of nitrate to ammonia. J. Alloys Compd..

[65] Zhang K., Sun P., Huang Y., Tang M., Zou X., Pan Z., Huo X., Wu J., Lin C., Sun Z. (2024). Electrochemical Nitrate Reduction to Ammonia on CuCo Nanowires at Practical Level. Adv. Funct. Mater..

[66] Huang L., Cheng L., Ma T., Zhang J.-J., Wu H., Su J., Song Y., Zhu H., Liu Q., Zhu M. (2023). Direct Synthesis of Ammonia from Nitrate on Amorphous Graphene with Near 100% Efficiency. Adv. Mater..

