# Supplementary material for: In Situ Formation of FeNi Nanoparticles on Polypyrrole Hydrogel for Efficient Electrocatalytic Nitrate Reduction to Ammonia
Source: Molecules. 2025 Mar 12;30(6):1271. doi: 10.3390/molecules30061271 (PMC11944797; doi:10.3390/molecules30061271)
Supplement: Supplementary file 1 [file molecules-30-01271-s001.zip › molecules-3440766-supplementary.pdf]

Supporting information

In Situ Formation of FeNi Nanoparticles on Polypyrrole Hydrogel for Efficient Electrocatalytic Nitrate Reduction to Ammonia

Lixia Li [1,†], Paihao Yan [1,†], Qinkai Guo [1,†], Dongxu Zhang [2], Chunliang Mao [3], Quan Yuan [1], Hongtao Sun [2],
Mingze Liu [2], Yanhong Liu [2,*] and Baodong Mao [2,*]

1 School of Environment and Safety Engineering, Jiangsu University, Zhenjiang 212013, China
2 School of Chemistry and Chemical Engineering, Jiangsu University, Zhenjiang 212013, China
3 College of Mechanical Engineering, Yanshan University, Qinhuangdao 066004, China
* Correspondence: liuyh@ujs.edu.cn (Y.L.); maobd@ujs.edu.cn (B.M.)
† These authors contributed equally to this work.

Debye–Scherrer formula (Formula S1):

$$D\ (\text{nm}) = \frac{K\times\lambda}{\beta\times\cos\theta} \tag{S1}$$

D (nm) is the crystalline size in nm. *K* is the Scherrer constant and takes the value 0.89. β can be calculated for the most intense peak for Fe/Ni-PPy nanocomposite. λ is the wavelength of Cu-kα1 (0.154060 nm). θ is the diffraction angle.

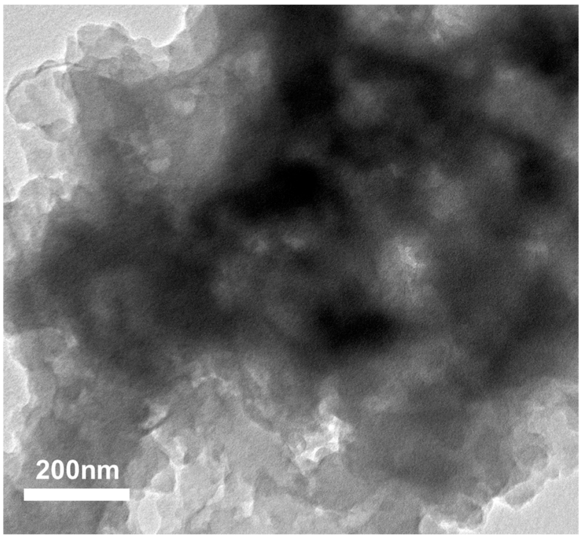

Figure S1. TEM image of Fe/Ni–PPy (Fe:Ni=1:1).

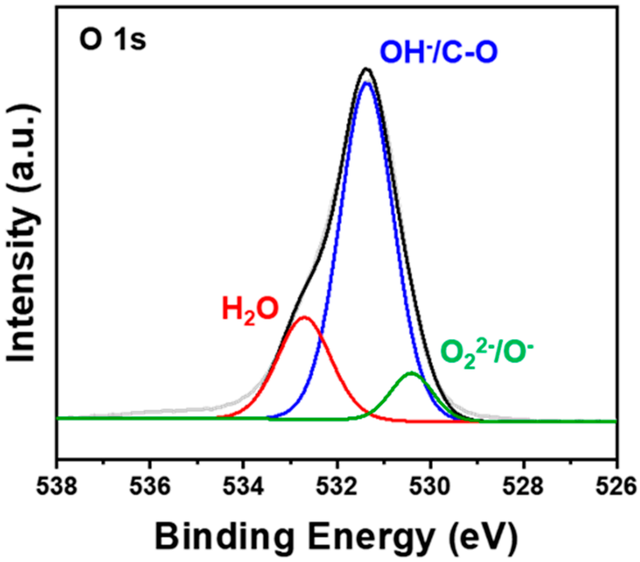

Figure S2. XPS spectrum of O 1s.

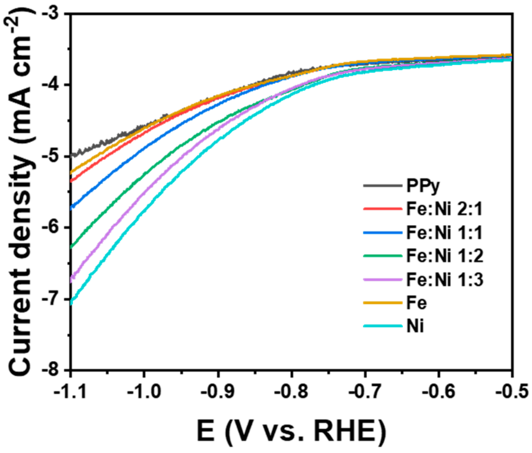

Figure S3. LSV curves of different catalysts with 0.5 M Na_2SO_4.

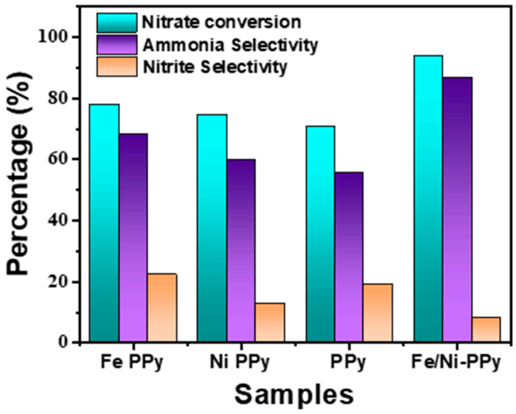

Figure S4. The performance comparison of different catalysts at −0.9 V vs. RHE for 1 h.

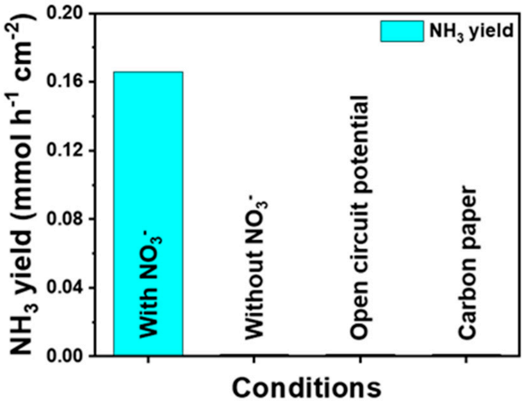

Figure S5. NH_3 yield in different conditions.

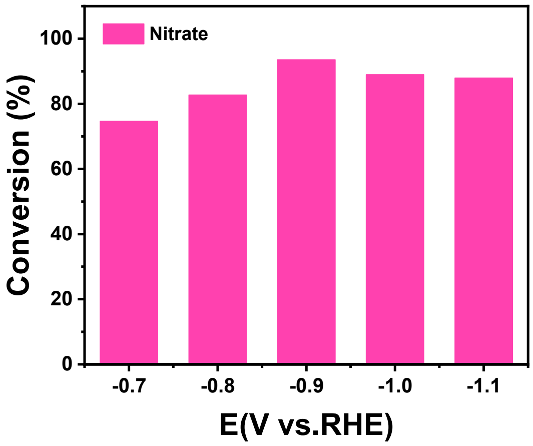

Figure S6. The conversion of NO_3^- by the Fe/Ni−PPy (Fe:Ni=1:1) at different potentials.

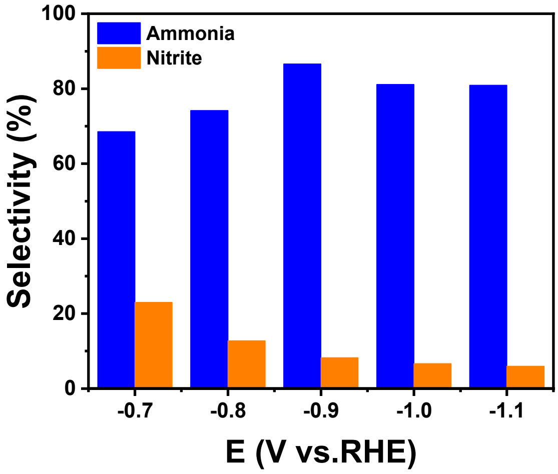

Figure S7. The selectivity of NH_3 and NO_2^- by the Fe/Ni−PPy (Fe:Ni=1:1) catalyst at different potentials.

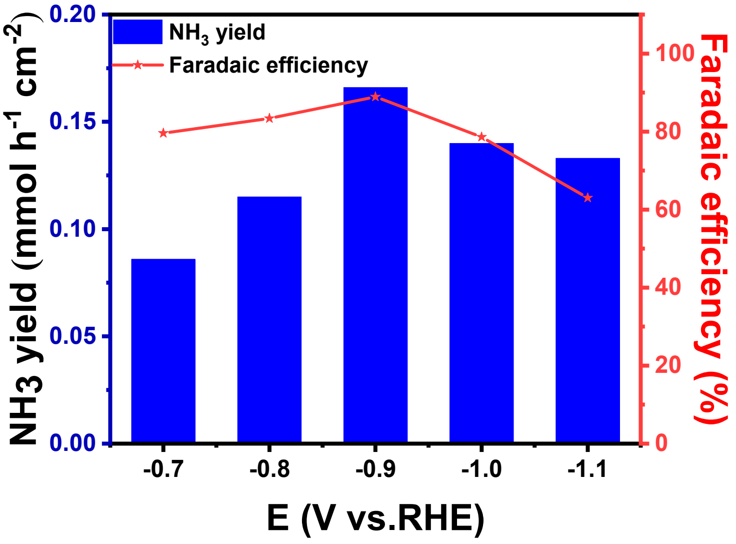

Figure S8. The NH_3 yield and FE by the Fe/Ni−PPy (Fe:Ni=1:1) catalyst at different potentials.

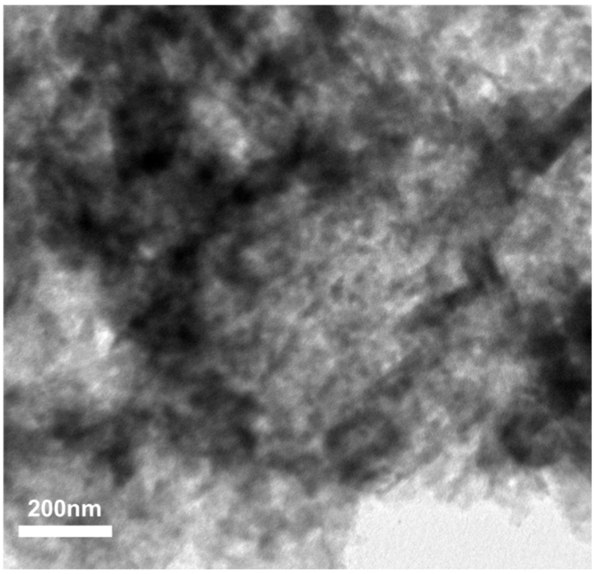

Figure S9. TEM image of Fe/Ni−PPy (Fe:Ni=1:1) after 6 continuous cycle tests.

Detection of nitrate

The electrolyte solution was removed from the cathode chamber and diluted 20 times to 5mL with 0.5 M Na_2SO_4, 0.1mL 1 M HCl and 0.01 mL 0.8 weight % sulfamic acid solution. After the mixture was left standing for 10 minutes, the absorption spectra in the wavelength range of 200-300nm were obtained by UV-2600 spectrophotometry. The absorbance value is calculated by the following formula: $A = A_{220nm} - 2A_{275nm}$. Different amounts of $NaNO_3$ were dissolved in 0.5M Na_2SO_4 solution to prepare standard solutions of different concentrations.

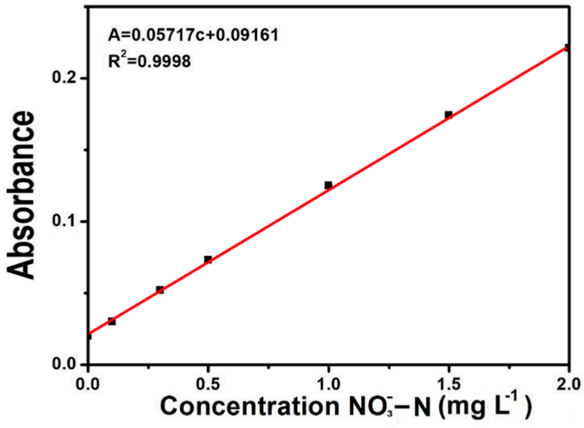

Figure S10. Standard curve of NO_3^-.

Detection of nitrite

The NO_2^- concentration was analysed using the Griess test. The Griess reagent was prepared by dissolving 0.1 g of N-(1-naphthyl) ethyldiamine dihydrochloride, 1.0 g of sulfonamide and 2.94 mL of H_3PO_4 in 50 mL of deionized H_2O. In a typical colorimetric assay, the Griess reagent (1.0 mL) was mixed with the nitrite-containing solution (1.0 mL) and H_2O (2.0 mL), and allowed to react at room temperature for 10 min, in which the sulfonamide reacts with the NO_2^- to form a diazonium salt and then further reacts with the amine to form an azo dye (magenta). The absorbance at 540 nm was measured by UV-2600 spectroscopy and the NO_2^- concentration was calculated. The fitting curve ($A = 1.52326c + 0.0342$, $R^2 = 0.9992$) shows good linear relation of absorbance value with NO_2^- concentration.

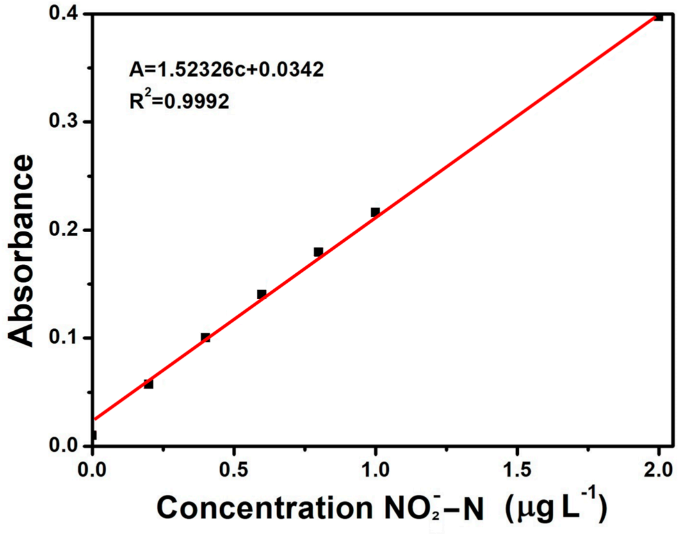

Figure S11. Standard curve of NO_2^-.

Detection of ammonia

Firstly, 2.0 mL electrolyte was taken from the cathodic side, and then 2.0 mL of 1 M NaOH solution containing 5% $C_7H_6O_3$ and 5% $C_6H_5Na_3O_7\cdot 2H_2O$ was added into this solution. Subsequently, 1.0 mL of 0.05 M NaClO and 0.2 mL of 1% $C_5FeN_6Na_2O\cdot 2H_2O$ were add into the above solution. After standing at room temperature for 1 h, the UV-2600 absorption spectrum was measured at a wavelength of 655 nm. The concentration-absorbance curves were calibrated using standard NH_3 solution with a series of concentrations. The fitting curve ($A = 0.19633c + 0.05212$, $R^2 = 0.998$) shows good linear relation of absorbance value with NH_3 concentration.

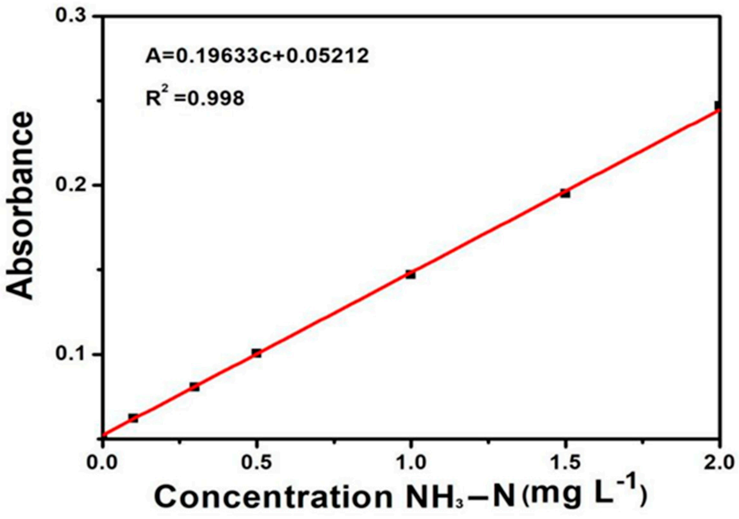

Figure S12. Standard curve of NH_3.
